# Hierarchical Self-Assembly of Metal-Ion-Modulated Chitosan Tubules

**DOI:** 10.1021/acs.langmuir.1c02097

**Published:** 2021-10-21

**Authors:** Pawan Kumar, Dániel Sebők, Ákos Kukovecz, Dezső Horváth, Ágota Tóth

**Affiliations:** † Department of Physical Chemistry and Materials Science, University of Szeged, Rerrich Béla tér 1., Szeged H-6720, Hungary; ‡ Department of Applied and Environmental Chemistry, University of Szeged, Rerrich Béla tér 1., Szeged H-6720, Hungary

## Abstract

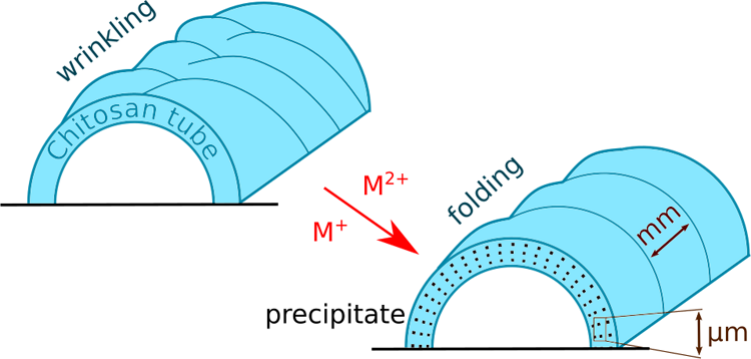

Soft materials such as gels or biological tissues can develop *via* self-assembly under chemo-mechanical forces. Here, we report the instantaneous formation of soft tubular structures with a two-level hierarchy by injecting a mixture of inorganic salt and chitosan (CS) solution from below into a reactor filled with alkaline solution. Folding and wrinkling instabilities occur on the originally smooth surface controlled by the salt composition and concentration. Liesegang-like precipitation patterns develop on the outer surface on a μm length scale in the presence of calcium chloride, while the precipitate particles are distributed evenly in the bulk as corroborated by X-ray μ-CT. On the other hand, barium hydroxide precipitates out only in the thin outer layer of the CS tubule when barium chloride is introduced into the CS solution. Independent of the concentration of the weakly interacting salt, an electric potential gradient across the CS membrane develops, which vanishes when the pH difference between the two sides of the membrane diminishes.

## Introduction

Structures with multi-level complexity are of utmost importance because they can be used as actuators,^1^ motors,^2^ adhesives,^3^ and so forth. They can be designed by a bottom-up approach starting from initial building blocks and creating the structure on a smaller length scale.^4,5^ Building an ordered polysaccharide polymer structure always gains attention as it enhances the functionality and performance of soft materials.^6,7^ The natural polysaccharide polymer chitosan (CS) is a potential candidate because of its biomedical applications^8,9^ and intrinsic properties such as biocompatibility,^10^ biodegradability,^11^ and bioactivity.^12^ The sol–gel transition leads to a multilayered CS polymer using special molds,^13^ stopping the gelation process^14,15^ or starting from appropriate salt compound.^16^

Monitoring the material ion composition, properties, and interaction strength helps in designing complex patterns of soft structures, which are utilized in a wide range of applications including stretchable electronics,^17^ biotemplating,^18^ biomineralization,^19^ bioactuation,^20^ 3-D printing,^21^ and so forth. In the last few years, CS interaction with metal ions has earned significant attention^22^ in waste water cleaning. It has been successfully applied to yield promising organized formations and structural modifications, where the strong affinity of CS to transition metal ions induces the orientation to layered transition and the weak affinity between alkaline earth metals and CS leads to composite gel structures.^23^ Our goal is to manipulate the patterns evolving at various length scales by changing the composition of metal salts added to the CS solution.

In order to acquire surface patterns, many elegant approaches have been developed mostly focusing on prefabricated gels. The swelling of hydrogels in solvents^24^ and in the presence of stimuli, like pH^25^ or photo-thermal effects,^26−29^ induces mechanical compression, resulting in the emergence of wrinkling instability. When strains develop on a soft substrate firmly attached to a rigid layer, the existing wrinkles are transformed into folds,^30−34^ which can also delaminate if adhesion between the soft and rigid material is weak.^35,36^ These types of patterns are abound in nature with a wide range of length scales: folds in the brain cortex^37^ evolve in μm size, while mm to cm size wrinkles appear on plants and human skin.^38^ Surface instabilities are also explored at geometries varying from planar sheets to cylindrical and spherical materials.^39^

Self-organized hollow tubules allow the separation of chemicals similar to the conduit structures of chemical gardens.^40−45^ In the emerging field of chemobrionics,^40,46^ the reaction between metal cations and silicate or other alkaline anions leads to precipitation of tubular membranes that produce chemical gradients between their two sides as was reported in iron mineral^47^ or sulfide-based membranes^48^ and silica chemical gardens.^49^ In deep-sea hydrothermal vents, black smoker chimney^50^ and hydrothermal fluid—sea water fuel cells^51^ generate electric energy. In prebiotic chemistry, the compartmentalization of chemical environments paves a significant contribution toward the origin of life.^52−54^

In the present work, we focus on multiscale instabilities arising during the boundary-aided growth of organic–inorganic tubules, where self-organized CS gel structures couple with self-assembled inorganic precipitates according to the distribution of supersaturation, giving rise to a spatial hierarchy. We not only characterize the evolving structures but also define the major factors determining them. Finally, the electrochemical description of the created membrane is provided for further applications.

## Experimental Section

Analytical grade reagents, medium molecular weight CS (Sigma-Aldrich 448877), CH_3_COOH (VWR, 99–100%), NaCl (Molar), KCl, CuCl_2_·2H_2_O (Reanal), CaCl_2_·2H_2_O, BaCl_2_·2H_2_O (VWR), and NaOH (Sigma-Aldrich, pellets) were used in the experiments. Solutions of CS 0.75 w/v % in 0.2 M CH_3_COOH and different concentrations of metal salts were prepared with doubly deionized water.

**Figure 1:**
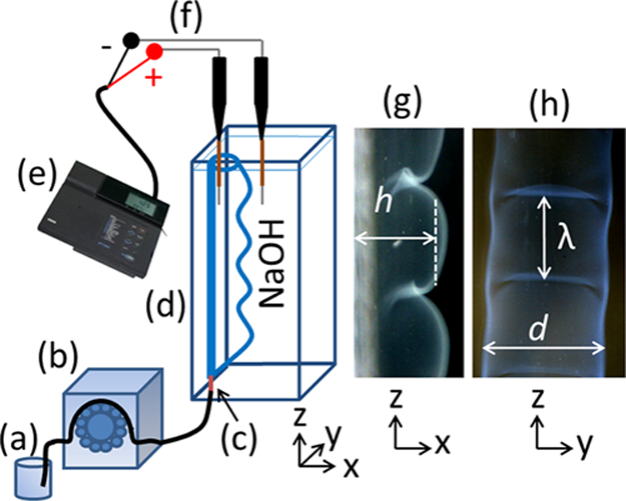
Schematic diagram of the experimental setup. (a) CS or CS–salt solution (b) peristaltic pump (c) injection inlet, (d) plexiglass cuvette, (e) multimeter, (f) platinum wires, (g) side view, and (h) front view of the tube. The definition of tube characteristics (*h*, *d*, and λ) is also shown.

CS or CS–salt solution was injected from below into the pool of NaOH solution (*c* = 0.75 M) using a twelve-roller peristaltic pump (Ismatec Reglo) through a Tygon tube (i.d. = 1.42 mm) with an inlet needle pinhole (i.d. = 0.6 mm) as shown in Figure 1. All experiments were performed at room temperature 23 ± 2 °C using a plexiglass cuvette with dimensions 1 cm × 2 cm × 10 cm (alkali and alkaline earth salts) or 1.5 × 2 × 3 cm^3^ (copper chloride). Images of the organized structures were recorded with a two-second temporal resolution using a Unibrain fire-i 630c camera with Vivitar extension tubes controlled by a computer.

For potential difference measurements, platinum wires having a diameter of 0.5 mm were polished with sandpaper no. 3000 before each experiment and covered with parafilm such that only their 6 mm long tip was exposed. By maintaining a 8 mm distance between them, the wires were inserted up to 1.8 cm (see Figure 1) into the electrolytes. The time-dependent electric potential inside the tube with respect to the outer electrolyte was recorded in a 5 s interval using a Thermo Orion 420 pH/mV meter connected to a computer.

For X-ray μCT measurements, Ca^2+^–, Ba^2+^–, and Cu^2+^–CS gel samples were carefully transferred from the alkaline solution into the empty sampling tube after 30 min. Then, the sampling tube was filled with deionized water. 3D characterization of each sample was obtained using X-ray μCT (Bruker SKYSCAN 2211 nanotomography, 55 kV accelerating voltage, 500 μA emission current). A total of 1390 images were extracted for 180° rotation with 0.15° rotation step and 10 μm pixel resolution using a 3 MP cooled Flat Panel camera (52 ms exposition time). The projection images were reconstructed using NRecon (SKYSCAN Bruker) software, and the volume-rendered 3D CT images were visualized using CTVox (SKYSCAN Bruker) software. Raman spectroscopic measurements were carried out with a Raman microscope (Senterra Bruker, 50x magnification, λ_exc_ = 785 nm, *P* = 10 mW).

To determine the gel thickness, a green laser beam (Roithner Lasertechnik, λ = 532 nm, *P* = 100 mW) passing through a lens (TechSpec) was projected vertically making a 90° angle with the camera.

## Results and Discussion

### Macroscale Patterns

When acidic polycation CS sol is injected into the alkaline solution, chemo-mechanical forces drive the boundary-assisted tubules, as discussed in our previous work for various types of CS gels.^55,56^ to various types of CS gels. The tube grows upward steadily on the glass wall, and compressive stress along the axial and circumferential direction produces the surface pattern: wrinkles deform far from the tube tip, while fold appears close to that.

**Figure 2:**
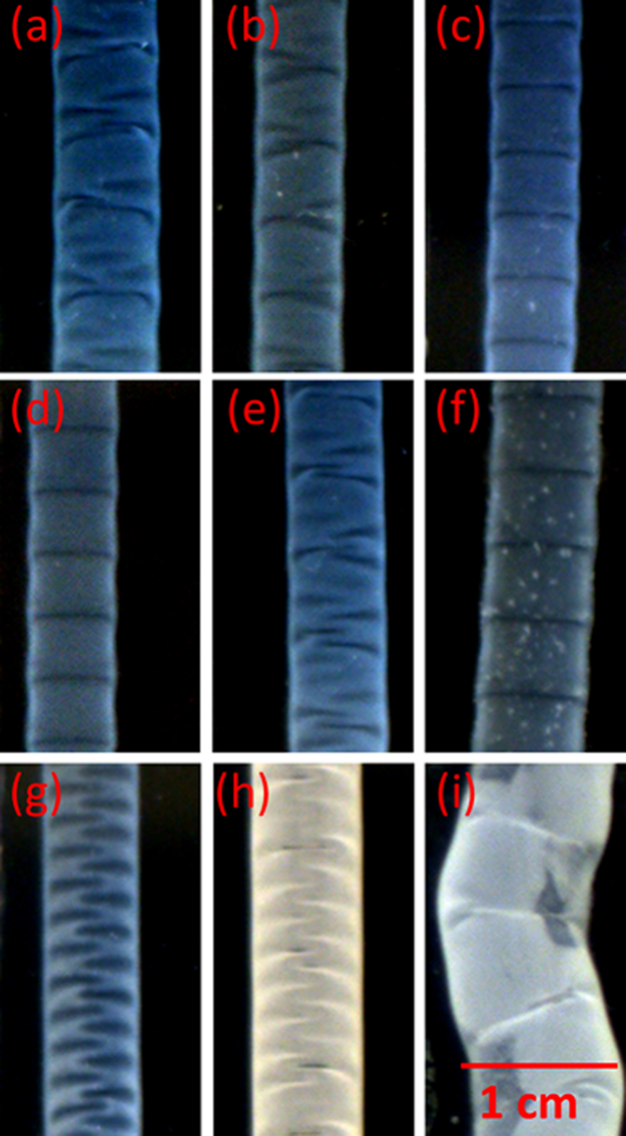
Surface patterning on the (a) CS at *t* = 54 s and (b–i) metal–CS tubes with [NaOH] = 0.75 M and *Q* = 1.01 mL min^–1^. The corresponding salt concentrations are (b) [NaCl] = 0.2 M, (c) 0.4 M, (d) [KCl] = 0.4 M, (e) [BaCl_2_] = 0.05 M, (f) 0.15 M, (g) [CaCl_2_] = 0.1 M, (h) 0.2 M, and (i) 0.3 M. The field view of all images is 2.37 × 1.27 cm^2^.

Patterns on the soft surface are monitored for a fixed alkaline concentration and injection rate when the ionic environment of the injecting CS sol is varied. An injection rate of *Q* = 1.01 mL min^–1^ was selected as a reference case because for the given alkaline concentration, coexisting modes of folds and wrinkles evolve on the pure CS tube,^56^ as shown in Figure 2a.

The addition of salts with monovalent cations, Na^+^ and K^+^, in different concentrations to the CS solution transforms the coexisting modes into folds (Figure 2b–d) with periodic deformations on the tubules with right angles to the direction of flow. The characteristic dimensions, such as diameter *d* and depth *h*, decrease, whereas the linear growth velocity *r*_l_ increases compared to that of pure CS tubes (see Table 1).

**Table 1:** Characteristic Properties of the CS and Metal–CS Tubules with [NaOH] = 0.75 M and *Q* = 1.01 mL min^–1^^a^

	*c* (M)	*d* (cm)	*h* (cm)	*r*_l_ (cm s^–1^)	*r*_V_ (cm^3^ min^–1^)	λ (cm)	shape
CS	0.75 w/v %	0.68 ± 0.01	0.32 ± 0.01	0.099 ± 0.001	1.01 ± 0.02		mixed
Na^+^	0.2	0.58 ± 0.01	0.28 ± 0.01	0.127 ± 0.001	0.96 ± 0.02	0.46 ± 0.01	F
Na^+^	0.3	0.58 ± 0.01	0.28 ± 0.01	0.126 ± 0.001	0.97 ± 0.02	0.43 ± 0.01	F
Na^+^	0.4	0.58 ± 0.01	0.29 ± 0.01	0.127 ± 0.001	1.00 ± 0.02	0.40 ± 0.01	F
K^+^	0.2	0.58 ± 0.01	0.27 ± 0.01	0.127 ± 0.001	0.95 ± 0.03	0.45 ± 0.01	F
K^+^	0.3	0.59 ± 0.01	0.28 ± 0.01	0.125 ± 0.001	0.98 ± 0.03	0.42 ± 0.01	F
K^+^	0.4	0.59 ± 0.01	0.29 ± 0.01	0.124 ± 0.001	0.98 ± 0.02	0.40 ± 0.01	F
Ca^2+^	0.1	0.61 ± 0.01	0.30 ± 0.01	0.115 ± 0.001	1.00 ± 0.03	0.21 ± 0.01	W
Ca^2+^	0.2	0.70 ± 0.01	0.34 ± 0.01	0.088 ± 0.001	0.99 ± 0.02	0.21 ± 0.01	W
Ca^2+^	0.3	0.92 ± 0.01	0.46 ± 0.01	0.050 ± 0.001	1.00 ± 0.01	0.56 ± 0.01	MF
Ba^2+^	0.05	0.63 ± 0.01	0.31 ± 0.01	0.109 ± 0.001	1.00 ± 0.02		mixed
Ba^2+^	0.1	0.65 ± 0.01	0.33 ± 0.01	0.101 ± 0.001	0.99 ± 0.02	0.47 ± 0.01	F
Ba^2+^	0.15	0.74 ± 0.01	0.36 ± 0.01	0.079 ± 0.001	0.98 ± 0.04	0.48 ± 0.01	F

a Symbol “F” represents the folding, “W” indicates the wrinkling instabilities, and “M” indicates meandering.

This is due to the salting-out effect^57^ because the screening of reactive protonated amine groups with Cl^–^ reduces the net electrostatic repulsion between the polymer chains. The increase in ionic strength therefore dominates the gel properties, and the tubules exhibit a slight decrease in the wavelength of the folding patterns (see Table 1). The increase in the alkali metal salt concentration, however, decreases the buoyancy effect, which provides an opposite influence on the tube growth characteristics governed by the flow. The balance between these two factors results in only insignificant changes in the tube diameter, depth, and linear growth rate.

Polycationic CS has weak affinity to not only alkali but also alkaline earth metal ions;^22^ therefore, the effect of Ba^2+^ and Ca^2+^ salts in the CS sol is also investigated. At low concentrations of barium ions, there is no change in the structure (Figure 2e), while at higher concentrations, a shift to the folding structure is observed (Figure 2f), similar to the case of Na^+^ and K^+^ contents. The addition of calcium ions represents a different scenario: wrinkling patterns (Figure 2g,h) with distinct formation of white precipitates are observed. A further increase in the alkaline earth salt concentration leads to the appearance of hierarchically structured patterns as the tubes meander along the wall, while surface instabilities appear only as secondary formations as shown for systems with calcium ions in Figure 2i (see Video S1 for the formation). The lower solubility of alkaline earth metal hydroxides is the key factor, which differentiates even Ca^2+^ and Ba^2+^. Upon contact, the encounter of OH^–^ produces Ba(OH)_2_ and Ca(OH)_2_ precipitates. The relatively high solubility product of barium hydroxide (p*K*_sp_ = 3.6^58^) results in only a negligible amount of precipitate, while tube contrast increases significantly for calcium hydroxide (p*K*_sp_ = 5.19) where substantial precipitation takes place.

Similar to alkali metal salts, at lower concentrations of alkaline earth metal salts, the linear velocity *r*_l_ increases because the tube diameter *d* and depth *h* decrease compared to those of the pure CS tube dimensions (Table 1) in accordance with the reduced repulsion between polymer chains due to the greater ionic strength. This is, on the other hand, accompanied by the decrease in the density difference between the two electrolytes, which weakens buoyant forces contributing to the tube formations and hence thickens the tube (see Table S1 in Supporting Information for the density differences). These two effects are again balanced by increasing the salt concentration with regards to the periodic patterns; hence, the wavelength of folding and wrinkling does not vary by increasing the alkaline earth concentrations of Ba^2+^ and Ca^2+^. Furthermore, precipitation takes place with the addition of the alkaline earth electrolytes, which also results in the increase of *d*.

Interestingly, independent of the composition, in the time scale of the experiments, the volume growth rates—calculated for half cylinders as *r*_V_ = π*dhr*_l_/4—of metal–CS and pure CS tubules are approximately equal to the injection rate *Q* (Table 1), indicating that there is no significant net liquid transfer across the organic–inorganic and organic membranes.^43^

We have also tested the effect of metal ions chemically interacting with CS. Our choice, copper ion, forms complexes with the amino groups; therefore, the strong affinity between Cu^2+^ and CS increases the cohesive force of the polymer chains and yields thicker tubes with short-wavelength buckling deformations (see Video S2).

**Figure 3:**
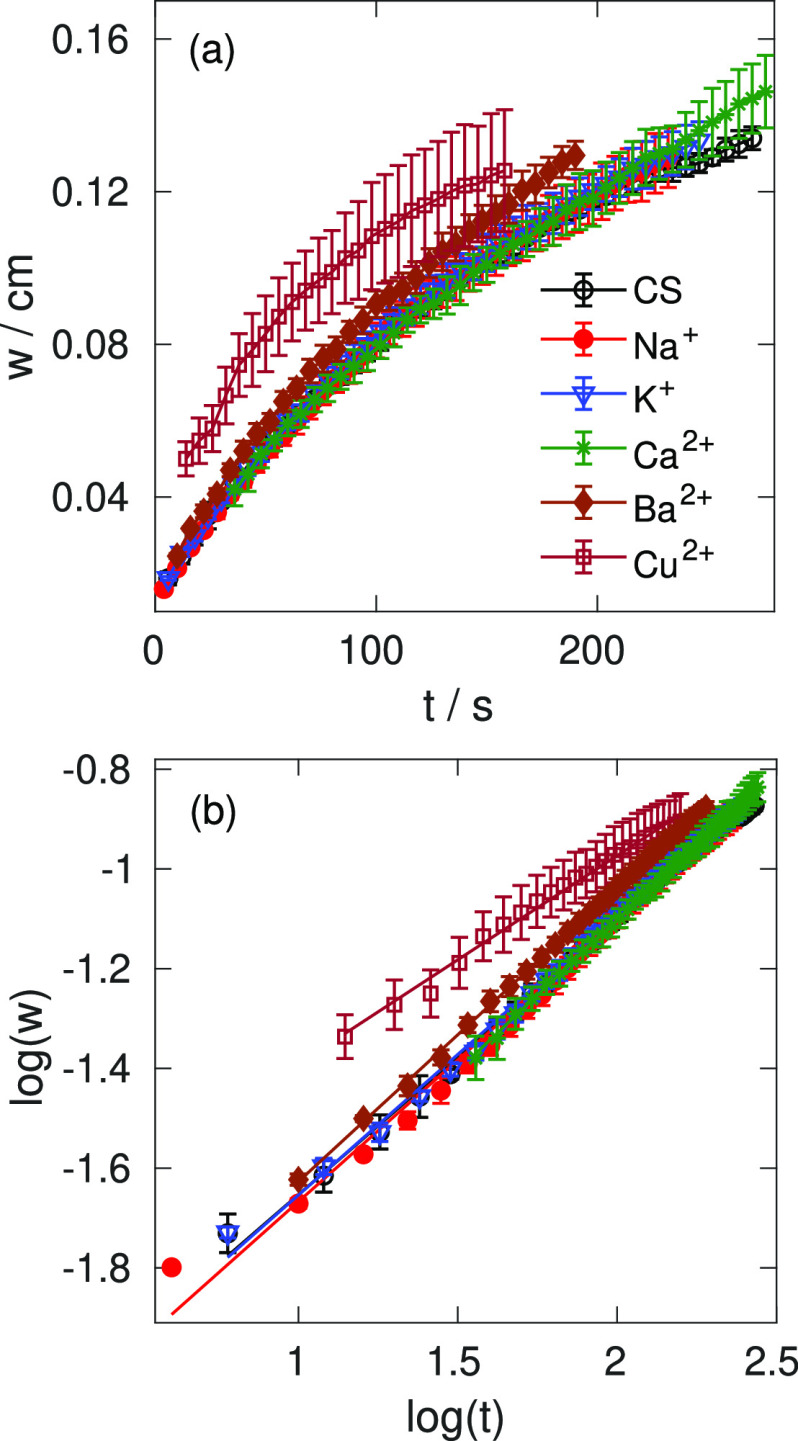
(a) Temporal evolution of CS and metal–CS membrane thickness at [NaOH] = 0.75 M, *Q* = 1.01 mL min^–1^; (b) corresponding logarithmic representation with the fitted lines.

The temporal growth of wall thickness *w* has been determined for the metal ion-containing tubes with highest concentrations as summarized in Figure 3.

The profiles of various membranes are similar, except for the copper(II)–CS gel where the complexation significantly alters the gel properties. The good overlap for the different scenarios and within the experimental error identical exponents (see α values in Table 2) suggests that the dominating driving force is the diffusion of hydroxide. The lower values of the scaling constants indicate the slow transportation of OH^–^ ions in the copper(II)–CS gel.

**Table 2:** Temporal Scaling Exponents (α) and Proportionality Constants (*k*) for CS and Metal–CS Membranes at [NaOH] = 0.75 M and *Q* = 1.01 mL min^–1^

name	[*c*] (M)	Α	10^5^*k* (cm^1/α^/s)
CS	0.75% w/v	0.56 ± 0.03	10.4 ± 1.8
Na^+^	0.4	0.57 ± 0.02	11.2 ± 1.0
K^+^	0.4	0.56 ± 0.01	11.1 ± 1.3
Ca^2+^	0.3	0.58 ± 0.03	10.9 ± 0.9
Ba^2+^	0.15	0.58 ± 0.02	14.7 ± 1.7
Cu^2+^	0.06	0.41 ± 0.03	3.9 ± 0.5

### Microstructure

**Figure 4:**
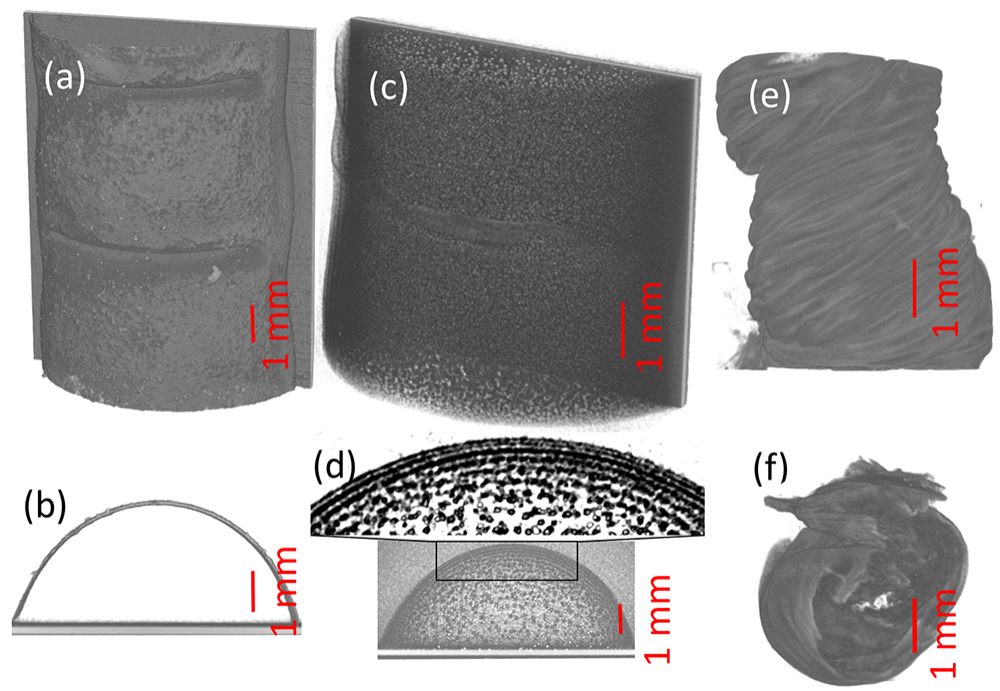
X-ray μCT measurements of the patterns with [NaOH] = 0.75 M, Q = 1.01 mL min^–1^ (a,b) [Ba^2+^] 0.15 M (c,d) [Ca^2+^] 0.3 M, and (e,f) [Cu^2+^] 0.06 M. The samples were removed from the cuvette 30 min after the 2 min-long injection was stopped and were used for immediate measurements.

The microscale patterns of the alkaline earth–CS gels have been characterized by X-ray micro-CT. The macropatterns of Ba^2+^–CS tubules are affected less by the addition of appropriate salts as illustrated in Figure 4a,b. Folding patterns and precipitation layers develop on the surface as precipitation takes place at the highest alkaline concentration region, that is, at the contact of the two electrolytes. The presence of the empty region in Figure 4b confirms that no precipitation takes place inside the tube. For Ca^2+^–CS gel, the solid particles seem to be homogeneously distributed as illustrated in Figure 4c,d but a close inspection reveals that the aggregation of solid particles forms periodic, Liesegang-like rings at the boundary (see Figure 4d enlarged section). The precipitation formation in the inner region is slower than the gelation process (see Figure S1) because of the smaller alkaline gradient and lower alkaline concentration resulting in the disruption of concentric bands. The precipitate is identified by Raman microscopy as a mixture of Ca(OH)_2_ and calcite; the latter is produced by the reaction with CO_2_ from the air.

The strong chemical interaction between Cu^2+^ and CS results in a highly buckled tube yielding a rigid membrane (see Figure 4e,f). These patterns resemble the hardening of the upper crust of a hot lava, where the lava underneath still flows.^59^

### Electrochemical Characteristics of the Membrane

**Figure 5:**
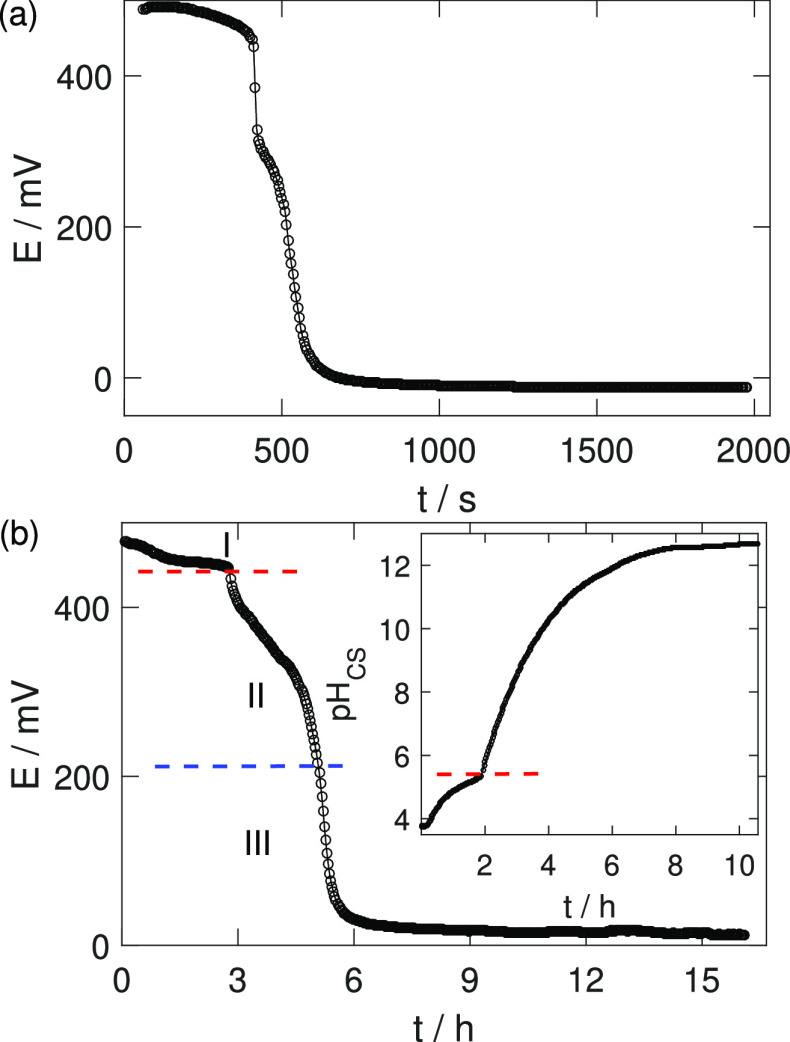
Electrochemical potential of (a) CS tubules and (b) CS membranes in a cuboid reactor. The inset figure displays the corresponding pH change in the CS compartment. The time when gelation reaches the appropriate electrode wire is marked by a red dashed line. The blue line indicates the time when the Pt wire is completely covered with gel.

Sol–gel transition triggers the boundary-aided CS tube formation that separates the acidic and basic electrolytes, and hence, a potential difference develops on the two sides of the membrane. We have placed two platinum wires in the solution such that in the proton-rich acidic medium, the Pt wire works as the cathode, while in the alkaline solution, it works as an anode. The drastic concentration gradient generates a potential difference of 484 ± 6 mV across the membrane, which changes with time due to the buildup of the membrane and the decreasing concentration gradient (Figure 5a). Following an initial slow decay, during which the wires are in the electrolytes, the electric potential exhibits two consecutive sharp steps before reaching the final ≈−12 mV in the time scale of the experiment.

For more insights, we performed an experiment with increased inner electrolyte volume, where a cuboid reactor is filled with an equal amount of acidic CS and basic NaOH solutions in different compartments, separated initially by a polyvinyl sheet (for experimental setup, see Figure S2). As we remove the sheet, CS gel forms immediately at the interface of the electrolytes. Besides the time-dependent potential gradient across the gel, the temporal changes in the pH of the electrolytes are measured separately. The potential difference evolves in three steps as a function of time as shown in Figure 5b, similar to the *in situ* tube formation but on a longer time scale because of the greater size. In region I, both wires are in the electrolytes. Region II begins when the sol–gel transition zone reaches the cathode wire. During the entire second region, some portion of the electrode is in contact with both the solution and the hydrogel. In region III, the electrode is completely covered with the hydrogel but the concentration gradient of the hydroxide ions exists. The pH of the CS solution increases with time due to the diffusion of hydroxide ions from the basic compartment (see inset of Figure 5b). The pH of the NaOH solution (pH_NaOH_) does not vary with time, and it is ≈13 even after 15 h. In the CS solution, the pH_CS_ = 3.75 ± 0.01 initially, which rises slowly corresponding to the first region of the potential. The contact of the gel with the pH electrode is marked by a red dashed line in Figure 5b, where pH_CS_ = 5.22 ± 0.12 and the corresponding potential difference is 456 ± 8 mV. The further steep pH rise indicates the gelation and causes a drastic fall in electric potential. After 15 h, pH_CS_ = 12.86 ± 0.03 is reached, which is approximately the same as the pH of the alkaline solution with 6 ± 3 mV potential difference.

Using the Nernst equation, the potential difference can be approximated as Δ*E* = *E*_CS_ – *E*_NaOH_ = 2.3*RT*/*F*(pH_NaOH_ – pH_CS_) because the pH gradient is the dominating factor with Pt wires immersed in aqueous solutions of nonelectroactive electrolytes in the presence of dissolved oxygen. The pH-driven potential difference is calculated to be Δ*E* = 451 mV in excellent agreement with the experimentally measured 450 mV.

The diffusion potential developed due to the presence of the ionic species can be calculated by solving the following dimensionless general balance equations^60^
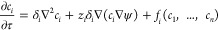
1
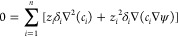
2where *c*_*i*_ represents the concentration of the *i*th species with charge *z*_*i*_ and relative diffusion coefficient δ_*i*_ = *D*_*i*_/*D*, τ = *t*/*t*_s_ is defined with *t*_s_ = 1 s, *f*_*i*_(*c*_1_, ..., *c*_*n*_) represents the chemical source/sink term related to the protonation/deprotonation reactions, and ψ = ϕ*F*/(*RT*) is the electric potential (see Supporting Information for details). The maximum potential difference ϕ is calculated to be 39 mV, which drops exponentially as a function of time (see Figure S3). Our analysis supports that Δ*E* largely depends upon the pH gradients and the final potential difference reached on the time scale matching that of the experiment is 0.2 mV. The time evolution of the electric potential and pH, however, differs because of the inevitable convection arising in the measurements. The electrochemical potential across the metal ion–CS membranes follows a similar profile to the CS membrane. For monovalent ions and the weakly interacting divalent ions, the potential difference is 420–450 mV for all the concentrations. The stronger interaction due to the complexation between copper ions and CS results in a 400 mV drop in the cell potential, and a longer potential evolution is obtained at greater concentration. Consequently, the temporal span of potential differences is shorter for thinner tubes and longer for the thicker tubes.

## Conclusions

We have shown surface patterns on tubular CS hydrogels, where coexisting modes transform into regular folds or wrinkles when metal salts were added into the CS solution. The increase in the monovalent ion concentration decreases the periodicity of folds, while no variation is observed in the characteristic properties. In contrast, the diameter and the depth of the tube increase for the alkaline earth metal chlorides. Wrinkles form at lower and intermediate calcium chloride concentrations, while at high concentrations, Liesegang-like concentric precipitation rings also appear on a smaller length scale. These variations are the results of the increase in ionic strength that changes the gel properties by decreasing the repulsion between the polymer chains. Copper ions have strong affinity to CS because of the strong amino complexation, which induces asymmetric wrinkle patterns as lava surfaces deform. Far-from-equilibrium organized tubules manifest the spontaneous separation of distinct electrolytes with electrochemical potential and pH gradients. The temporal evolution of the cell potential on the two sides of the membrane is affected by the *in situ* thickening of the wall, accompanied by the movement of the hydroxide ions. The present work provides a controlled framework of the metal ion–CS hierarchical self-assembly, which can be useful for bioinspired material applications of energy generation as thin films for fuel cells or lithium ion batteries.^61^

## Supporting Information Available

Macro- and microscale characterization and calculational details on estimation of diffusional potentials across the membrane (PDF)
Folding patterns on Ca-CS tube (AVI)
Deformations on the Cu-CS membrane (AVI)

## Supplementary Material

la1c02097_si_001.pdf

la1c02097_si_002.avi

la1c02097_si_003.avi
