# Supplementary material for: Hierarchical Self-Assembly of Metal-Ion-Modulated Chitosan Tubules
Source: Langmuir. 2021 Oct 21;37(43):12690–6. doi: 10.1021/acs.langmuir.1c02097 (PMC8567419; doi:10.1021/acs.langmuir.1c02097)
Supplement: Supplementary file 1 — la1c02097_si_001.pdf [file la1c02097_si_001.pdf]

Supporting Information: Hierarchical self-assembly of metal-ion modulated chitosan tubules

Pawan Kumar,† Dániel Sebők,‡ Ákos Kukovecz,‡ Dezső Horváth,‡ and Ágota Tóth*,†

†*Department of Physical Chemistry and Materials Science, University of Szeged, Rerrich Béla tér 1., Szeged, H-6720, Hungary.*

‡*Department of Applied and Environmental Chemistry, University of Szeged, Rerrich Béla tér 1., Szeged, H-6720, Hungary.*

E-mail: atoth@chem.u-szeged.hu

Contents

1 Macro- and microscale characterization **S2**

2 Estimation of diffusion potential across the membrane **S4**

3 Supporting Videos **S6**

References **S6**

1 Macro- and microscale characterization

The density of the solutions was measured with a density meter (Anton Paar DMA-550) .

Table S1: Density of the CS solution and CS-salt solutions where $\delta\rho = (\rho_{NaOH}$ - $\rho)$ represents the density difference between the alkaline and the injected solution with $\rho_{NaOH} = 1.0292\ \mathrm{g\,cm^{-3}}$.

Reagent	c [M]	ρ (g/cm^3)	$\delta\rho$ (g/cm^3)
CS	0.75 w/V %	1.0013	0.0279
K^+	0.20	1.0112	0.0180
K^+	0.30	1.0156	0.0136
K^+	0.40	1.0199	0.0093
Na^+	0.20	1.0096	0.0196
Na^+	0.30	1.0139	0.0153
Na^+	0.40	1.0171	0.0121
Ca^{2+}	0.10	1.0110	0.0182
Ca^{2+}	0.20	1.0191	0.0101
Ca^{2+}	0.30	1.0288	0.0004
Ba^{2+}	0.05	1.0110	0.0182
Ba^{2+}	0.10	1.0197	0.0095
Ba^{2+}	0.15	1.0285	0.0007
Cu^{2+}	0.02	1.0038	0.0254
Cu^{2+}	0.04	1.0062	0.0230
Cu^{2+}	0.06	1.0086	0.0206

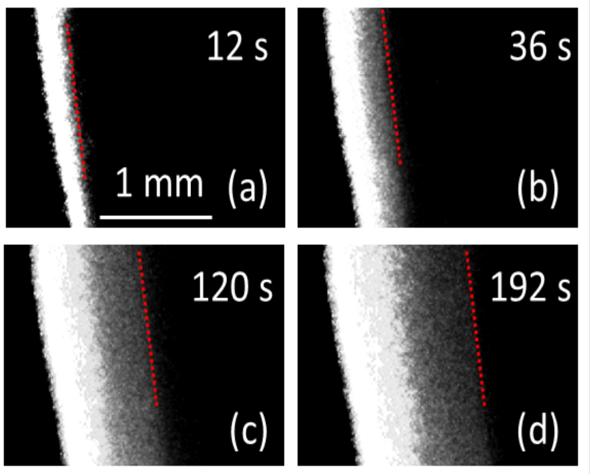

Figure S1: (a-d) Temporal evolution of Ca(II)-CS tube wall thickness w with $[Ca^{2+}] = 0.3$ M, [NaOH] = 0.75 M, and $Q = 1.01$ mL min^{-1}. Bright white region indicates the precipitation. The dotted line represents the inner boundary of the tube. Field view is 0.25×0.17 cm^2.

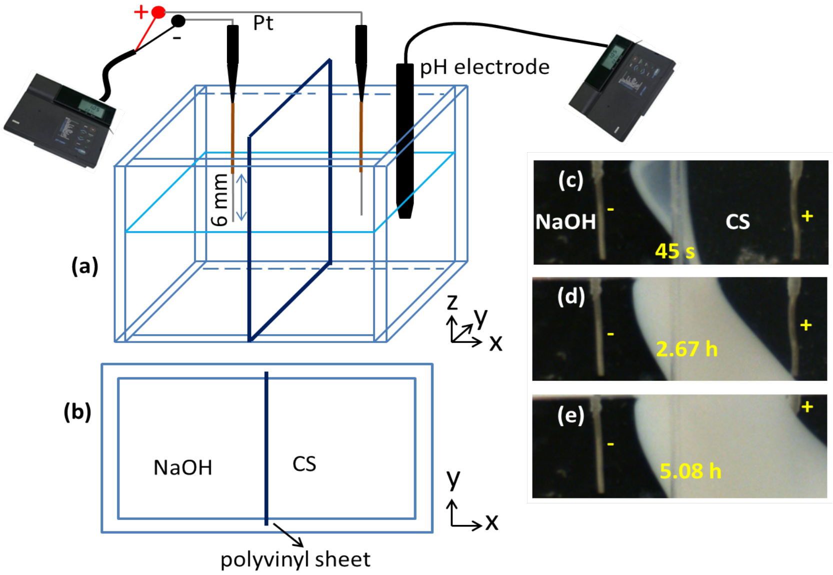

Figure S2: (a) Scheme of the experimental setup with a plexiglass cuboid reactor ($4 \times 2 \times 3$ cm^3) and (b) top view of the setup. A thin polyvinyl sheet (0.2 mm thickness) separates the CS and NaOH solutions into two compartments. Polished platinum wires of 0.5 mm diameter are covered with parafilm such that only 0.6 cm length is exposed. The potential differences and pH of both electrolytes are measured with a pH/mV meter. Images of the gel formation when (c) electrodes are in the electrolytes, (d) gel touches the cathode electrode, and (e) cathode electrode is fully covered with the gel. Chemical composition: [NaOH] = 0.75 M, [CS] = 0.75 w/V% in 0.2 M CH_3COOH.

2 Estimation of diffusion potential across the membrane

In our experimental study, chitosan membrane separates the two electrolytes in the different compartments with the following reversible chemical reactions

$$
\begin{aligned}
H^+ + OH^- &\rightleftharpoons H_2O \qquad \text{(S1)} \\
HAc &\rightleftharpoons H^+ + Ac^- \qquad \text{(S2)} \\
CS{-}NH_2 + H^+ &\rightleftharpoons CS{-}NH_3{}^+ \qquad \text{(S3)}
\end{aligned}
$$

where HAc represents acetic acid, Ac^- acetate ions, and CS-NH_2 chitosan. The values of the dimensionless rate constants are for Eqn. (S1) $k_1 = 10^9$ and $k_{-1} = 10^{-5}$, for Eqn. (S2) $k_2 = 10^4$ and $k_{-2} = 10^9$, while for Eqn. (S3) $k_3 = 10^9$ and $k_{-3} = 1$, for the corresponding forward and backward reaction, respectively.

The dimensionless governing equations[S1] are used in the form of

$$
\begin{aligned}
\frac{\partial c_i}{\partial \tau} &= \delta_i \nabla^2(c_i) + z_i \delta_i \nabla(c_i \nabla\psi) + f_i(c_1, ..., c_n) \qquad \text{(S4)} \\
0 &= \sum_{i=1}^{n} [z_i \delta_i \nabla^2(c_i) + z_i{}^2 \delta_i \nabla(c_i \nabla\psi)] \qquad \text{(S5)}
\end{aligned}
$$

where c_i represents the dimensionless concentration of the ith species relative to the standard concentration c^0 with z_i charge and δ_i dimensionless diffusion coefficients and $\tau = t/t_0$ with $t_0 = 1$s.

Based on the reactions given in Eqns (S1)–(S3), the rate equations of the different species

are described as

$$f(c_1) = -k_1c_1c_2 + k_2 + k_3c_3 - k_4c_1c_4 - k_5c_5c_1 + k_6c_6 \quad (S6)$$

$$f(c_2) = -k_1c_1c_2 + k_2 \quad (S7)$$

$$f(c_3) = -k_3c_3 + k_4c_1c_4 \quad (S8)$$

$$f(c_4) = k_3c_3 - k_4c_1c_4 \quad (S9)$$

$$f(c_5) = -k_5c_5c_1 + k_6c_6 \quad (S10)$$

$$f(c_6) = k_5c_5c_1 - k_6c_6 \quad (S11)$$

$$f(c_7) = 0 \quad (S12)$$

with dimensionless concentrations c_1, c_2, c_3, c_4, c_5, c_6, and c_7 corresponding to the concentrations of H^+, OH^-, HAc, Ac^-, CS-NH_2, CS-$NH_3{}^+$ and Na^+ species, respectively.

To mimic the experiments, the partial differential equations are solved in 1D, where the one dimensional grid system of 1000 grids is divided into two compartments, chamber A with 300 and chamber B with 700 grids with spacing of 10^{-3} cm mimicking the 1.0 cm cuvette. For the calculations, CVODE[S2] solver with the Backwards Differentiation Formula (BDF) is used from the SUNDIALS[S3] package with initial conditions given in Table S2, relative tolerances of 10^{-12}, and absolute tolerances of 10^{-16}.

Table S2: The species with their charge z, dimensionless diffusion coefficient δ and the initial dimensionless concentrations c_i in chambers A and B

No.	Name	z	$10^5 D(\mathrm{cm}^2/\mathrm{s})$	c(chamber A)	c(chamber B)
1	H^+	1	13.7	0	0.112
2	OH^-	-1	7.5	0.75	0
3	HAc	0	1.6	0	0.044
4	Ac^-	-1	1.6	0	0.156
5	CS-NH_2	0	2.0	0	0
6	CS-NH_3^+	1	0	0	0.044
7	Na^+	1	0	0.75	0

We have found that the temporal evolution of the potential difference ϕ decreases expo-

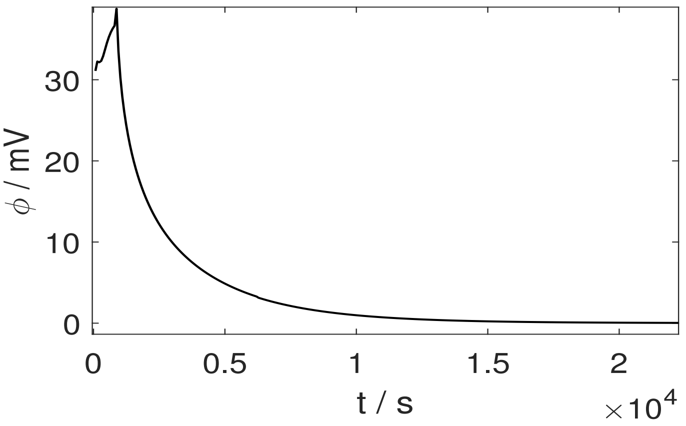

Figure S3: Temporal evolution of the diffusion potential.

nentially (see Figure S3) with a maximum of 38 mV diffusion potential. We have repeated the calculations by introducing monovalent salt in different concentrations. The tendency has not changed, only the maximum value of the diffusion potential has decreased to 20 mV on increasing the concentration of NaCl to 0.3 M.

3 Supporting Videos

VideoS1: Folding patterns on Ca-CS tube, shown at 10x real time. The experimental conditions are [Ca^{+2}]: 0.3 M in 0.75 w/V% CS, Q: 1.01 mL min^{-1}, [NaOH]: 0.75 M.

VideoS2: Deformations on the Cu-CS membrane, shown at 12x real time . The experimental conditions are [Cu^{+2}]: 0.06 M in 0.75 w/V% CS, Q: 1.01 mL min^{-1}, [NaOH]: 0.75 M.

References

(S1) Virányi, Z.; Tóth, Á.; Horváth, D. Diffusion-driven pattern formation in ionic chemical solutions. *Phys. Rev. Lett.* **2008**, *100*, 088301.

(S2) Cohen, S. D.; Hindmarsh, A. C.; Dubois, P. F. CVODE, a stiff/nonstiff ODE solver in C. *Computers in Physics* **1996**, *10*, 138–143.

(S3) Hindmarsh, A. C.; Brown, P. N.; Grant, K. E.; Lee, S. L.; Serban, R.; Shumaker, D. E.; Woodward, C. S. SUNDIALS: Suite of nonlinear and differential/algebraic equation solvers. *ACM Trans. Math. Softw.* **2005**, *31*, 363–396.
